# Hypoxia Affects the Structure of Breast Cancer Cell-Derived Matrix to Support Angiogenic Responses of Endothelial Cells

**DOI:** 10.4172/2157-2518.S13-005

**Published:** 2013-06-15

**Authors:** Abigail Hielscher, Connie Qiu, Josh Porterfield, Quinton Smith, Sharon Gerecht

**Affiliations:** 1 Department of Chemical and Biomolecular Engineering, Johns Hopkins University, Baltimore, MD 21218, USA; 2 Johns Hopkins Physical Sciences-Oncology Center, Johns Hopkins University, Baltimore, MD 21218, USA; 3 Institute for NanoBioTechnology, Johns Hopkins University, Baltimore, MD 21218, USA

**Keywords:** Hypoxia, Extracellular matrix, Angiogenesis

## Abstract

Hypoxia, a common feature of the tumor environment and participant in tumor progression, is known to alter gene and protein expression of several Extracellular Matrix (ECM) proteins, many of which have roles in angiogenesis. Previously, we reported that ECM deposited from co-cultures of Neonatal Fibroblasts (NuFF) with breast cancer cells, supported 3-dimensional vascular morphogenesis. Here, we sought to characterize the hypoxic ECM and to identify whether the deposited ECM induce angiogenic responses in Endothelial Cells (ECs). NuFF and MDA-MB-231 breast cancer cells were co-cultured, subjected to alternating cycles of 24 hours of 1% (hypoxia) and 21% (atmospheric) oxygen and de-cellularized for analyses of deposited ECM. We report differences in mRNA expression profiles of matrix proteins and crosslinking enzymes relevant to angiogenesis in hypoxia-exposed co-cultures. Interestingly, overt differences in the expression of ECM proteins were not detected in the de-cellularized ECM; however, up-regulation of the cell-binding fragment of fibronecin was observed in the conditioned media of hypoxic co-cultures. Ultrastructure analyses of the de-cellularized ECM revealed differences in fiber morphology with hypoxic fibers more compact and aligned, occupying a greater percent area and having larger diameter fibers than atmospheric ECM. Examining the effect of hypoxic ECM on angiogenic responses of ECs, morphological differences in Capillary-Like Structures (CLS) formed atop de-cellularized hypoxic and atmospheric ECM were not evident. Interestingly, we found that hypoxic ECM regulated the expression of angiogenic factors and matrix metalloproteinases in CLS. Overall, we report that *in vitro*, hypoxia does not alter the composition of the ECM deposited by co-cultures of NuFF/MDA-MB-231, but rather alters fiber morphology, and induces vascular expression of angiogenic growth factors and metalloproteinases. Taken together, these results have important implications for understanding how the hypoxic matrix may regulate angiogenesis in tumors.

## Introduction

Hypoxia is a pervasive presence within the tumor microenvironment, contributing to tumor angiogenesis, metastasis and resistance to chemotherapeutic drugs [1]. With regard to angiogenesis, it is widely known that low oxygen tension within the tumor microenvironment stimulates the growth of new vessels, in part through activation of pro-angiogenic cytokines such as Vascular Endothelial Growth Factor (VEGF) and Angiopoietin 2 (ANG2) [2,3]. Breast cancer was shown that hypoxia significantly increased the invasive capabilities of lymphatic Endothelial Cells (ECs) into a reconstituted basement membrane matrix when the cells were cultured in the presence of the metastatic breast cancer cell line MDA-MB-231 [4]. While these and other studies illustrate a role for hypoxia in the activation of ECs during vascular morphogenesis, less is known about the involvement of the Extracellular Matrix (ECM) during hypoxia-driven angiogenesis.

The ECM is a network of proteins and proteoglycans which supports diverse cellular functions including angiogenesis [5]. Here, the ECM sequesters the pro-angiogenic factors, which promote EC activation and migration into the stromal space, and provides a supportive framework from which ECs proliferate, migrate, align and assemble into new vessels [6,7]. Recently, we and others have reported that fibroblasts and co-cultures of fibroblasts with breast cancer cells deposit ECM, which is sufficient for directing the assembly of 3-dimensional (3D) vascular structures [8,9]. Apart from the collective ECM, others have elucidated the unique and overlapping functions of individual ECM proteins as they relate to angiogenesis. For instance, it has been shown that fibronectin supports vascular survival, migration and proliferation [10-12] while collagen IV promotes vascular elongation and survival [13] and laminin induces vascular elongation [14]. It would appear from these studies that individual proteins direct different vascular responses, which together, permit organized vascular morphogenesis.

Numerous studies point to the role of hypoxia in altering the expression of ECM proteins. For instance, we have recently shown that hypoxia increases the expression of collagens I and IV, laminin and fibronectin deposition from ECs [15]. Similarly, others have shown using fibroblasts and animal models that hypoxia increases collagen I mRNA expression, synthesis and deposition [16-20], fibronectin protein expression [21,22], collagen IV mRNA expression and secretion [21,23], and tenascin-C mRNA and protein expression [24]. In addition to these, hypoxia has also been reported to influence the expression of prolyl hydroxylases and lysyl oxidases (LOX), enzymes which stabilize and promote crosslinking of collagens and elastins in the ECM [25]. These findings highlight the putative role of hypoxia in regulating the expression and stability of ECM proteins in cells.

Changes in the composition of the ECM influence angiogenesis through activation and/or repression of various genes and proteolytic enzymes in ECs [26,27]. For instance, when ECs were grown in a 3D collagen gel as opposed to Matrigel, the ECs expressed two well-characterized pro-angiogenic matrix metalloproteinases (MMPs), MT1-MMP and MMP2, proteolytic enzymes which degrade the ECM and allow EC invasion and network formation in the stromal space [27,28]. Later it was subsequently shown that these MMPs played a major role in vascular network formation in 3D matrices [29,30]. Given the role of the ECM in effecting EC behaviors, it is important to understand whether hypoxia-induced changes in the ECM alter EC responses and angiogenesis.

While several studies have independently reported on the ECM’s participation in angiogenesis and the role of hypoxia in altering ECM properties, none, to our knowledge, has investigated angiogenesis in the context of hypoxia-induced changes in the ECM. We hypothesized that hypoxia induces changes in deposited ECM properties which in turn regulates EC responses and morphogenesis. Toward this, we have evaluated how the properties of ECM, deposited by co-cultures of human Neonatal Foreskin Fibroblasts (NuFF) with MDA-MB-231 breast cancer cells, are modified following exposure to either hypoxia or atmospheric conditions, and whether these influence vascular morphogenesis of Human Umbilical Vein Endothelial Cells (HUVECs).

## Materials and Methods

### Cell Lines

The MDA-MB-231 (brevity, MDA231) breast cancer cell line was a gift from the National Cancer Institute Physical Sciences-Oncology Center (National Institutes of Health; Bethesda, MD) and was obtained through the laboratory of Dr. Thea Tlsty (University of California San Francisco, San Francisco, CA). MDA231 cells were maintained in DMEM supplemented with 10% vol/vol FBS (Atlanta Biologicals; Lawrenceville, GA). The human neonatal foreskin fibroblast (NuFF) cell line was obtained from Global Stem (Rockville, MD) at passage 9 and was used up to passage 28 for all experiments. NuFF cells were cultured in DMEM supplemented with 10% vol/vol heat inactivated FBS (Invitrogen, Carlsbad, CA). Human Umbilical Vein Endothelial Cells (HUVECs; Passage 5 Promocell, Heidelberg, Germany) were cultured in EGM media supplemented with 2% FBS (both from Promocell). Media was replaced every 2-3 days and cells were passaged after reaching 80-90% confluency using 0.25% trypsin EDTA (Sigma, Allentown, PA) for NuFF and MDA231 or 0.05% typsin EDTA for HUVECs. All cell lines were maintained at 37°C in a humidified atmosphere containing 5% CO_2_.

### Co-Cultures

NuFF and MDA231 were co-cultured at a 1:1 ratio as previously described [8]. Cell numbers did not exceed a total of 1.4 × 10^5^ cells/cm^2^ for co-cultures and monocultures of NuFF at the time of seeding. Co-cultures and NuFF monocultures were maintained at 37°C in a humidified atmosphere containing 5% CO_2_ for 9 days with media changes every 2-3 days.

### Hypoxia

Co-cultures of NuFF/MDA231 and monocultures of NuFF were exposed to 24-hour alternating cycles of 1% O_2_ and 21% O_2_ for a total of 9 days. The de-cellularized matrix obtained from each condition is referred to as hypoxic or atmospheric ECM. As a control, NuFF/MDA231 co-cultures and NuFF monocultures were maintained in 21% O_2_ for 9 days. For hypoxia, we followed previous procedures [15,31]. In short, cultures were placed in a modular hypoxia chamber (Billups-Rothenberg; Del Mar, CA) containing a Petri dish of deionized water, used to maintain a humidified atmosphere. The chamber was flushed 3 times for 3 minutes each with oxygen at a gas mixture of 1% O_2_, 5% CO_2_-N_2_ balance. The chamber with cells was maintained in a humidified atmosphere at 37°C.

### De-cellularization and seeding of HUVECs

Isolation of co-culture or monoculture ECM and seeding of HUVECs were performed as previously described [8,9]. For analyses of the influence of hypoxia or atmospheric ECM, HUVECs were seeded on de-cellularized ECM obtained from hypoxia (1% O_2_) and atmospheric (21% O_2_) NuFF/MDA231 co-cultures or NuFF monocultures. After 24 hours in culture, vascular structures were either fixed or stained for visualization of vascular organization, morphology and abundance or RNA was collected for gene expression analyses.

### Immunofluorescence

De-cellularized ECM and vascular structures grown atop de-cellularized ECM were fixed in 3.7% paraformaldehyde for 30 minutes. Cells were permeabilized in 0.1% triton-X for 10 minutes. Cells and ECM were blocked in 1% bovine serum albumin (BSA) for 30 minutes and incubated with primary antibody for one hour followed by a one hour incubation in secondary antibodies (Table 1). Nuclei were stained using DAPI for 10 minutes. Two washes in 1X PBS were used after all steps. All immunolabeled samples were mounted using Dako fluorescent mounting media and visualized using fluorescence microscopy (Olympus BX60, Olympus, Center Valley, PA) or confocal microscopy (LSM 510 Meta, Carl Zeiss).

### Western blot

De-cellularized hypoxic and atmospheric ECM (day 9) and HUVECs grown for 24 hours on hypoxic and atmospheric ECM were solubilized or lysed and quantified for protein content as previously described [8]. For analysis of secreted ECM proteins, conditioned media was collected from co-cultures after 3, 5 and 9 days in hypoxia or atmospheric conditions and spun down at 4°C for one hour at 20,000 x g. Ponceau S (Amresco, Solon, OH) was utilized as a loading control for analyses of protein expression in conditioned media. A total of 30 μl of conditioned media (15 μl of laemmli buffer; 15 μl of conditioned media), 10 μg of vascular cell lysate and 50 μg of de-cellularized matrix from hypoxia and atmospheric cultures was loaded per well into a 4-20% SDS PAGE gel (BioRad, Hercules, CA) and ran as previously described [8]. Blots were visualized using the ChemiDocTM XRS+ System (Biorad). Images were acquired using Biorad Quantity One™ software.

### Enzyme zymography

HUVECs were seeded on de-cellularized hypoxic or atmospheric ECM for 24hrs. After 12 hours of growth in EGM media supplemented with 2% serum, the media was exchanged to serum free EGM media for the remaining 12 hours of vascular assembly. Supernatant was collected after 24hrs, spun down at 20,000 x g for 10 minutes at 4°C and stored at -80°C until analysis. The supernatant was diluted 1:1 with laemmli buffer without addition of reducing agents. A volume of 30μl was loaded on a 12% casein gel (Invitrogen) for MMP1 or a 10% gelatin gel (Invitrogen) for MMPs 2 and 9. The gels were run at 150V for 1.5hours in SDS running buffer, followed by a series of four 15 minute washes in1X renaturation buffer (Invitrogen). The gels were transferred to 1X Denaturation Buffer (Invitrogen) for 15 minutes with gentle shaking and then placed at 37°C for incubation overnight. The following day, the gels were fixed in 50% methanol and 10% acetic acid for 30 minutes and stained in 0.02% Coomassie (Sigma) in 50% methanol and 10% acetic acid for 2 hours. The gels were de-stained for 1-2 hours in 20% methanol, 10% acetic acid solution and transferred to deionized H_2_O. Gels were visualized using ChemiDoc™ XRS+ System (Biorad, Hercules, CA) and imaged using Biorad Quantity One™ software (Biorad).

### qRT-PCR

Two-step qRT-PCR was performed on cDNA from NuFF/MDA231 co-cultures grown in hypoxia or atmospheric conditions or on vascular structures grown on hypoxic or atmospheric ECM for 24 hours as previously described [8,15,31]. The TaqMan Universal PCR Master Mix and Gene Expression Assay (Applied Biosystems, Foster City, CA) was used for analysis of collagen Ia1, Lysyl Oxidase (LOX), Lysyl Oxidase-Like Protein *2* (LOXL2), Vascular Endothelial Growth Factor *A* (VEGFA), Vascular Endothelial Growth Factor Receptor 2 (VEGFR2), Fibronectin, Angiopoietin 1 (ANG1), angiopoietin *2* (ANG2), matrix metalloproteinases (MMPs) 1,2, and 9 and *MT1-MMP* (MMP 14) according to the manufacturer’s instructions. The TaqMan PCR step was performed with an Applied Biosystems StepOne Real-Time PCR System (Applied Biosystems), following the manufacturer’s instructions. The relative expression of each gene was normalized to the amount of β-actin or GAPDH in the same cDNA through use of the standard curve method described by the manufacturer. Significant changes in control gene expression were not observed during the course of experiments. For each primer set, the comparative computerized tomography method (Applied Biosystems) was used to calculate amplification differences between hypoxia and atmospheric-exposed NuFF/MDA231 and vascular structures on de-cellularized ECM.

### Scanning electron microscopy

De-cellularized ECM from NuFF/MDA231 co-cultures grown in atmospheric or hypoxia were fixed and prepared for scanning electron microscopy as previously described [8].

### Image analysis of vascular structures and ultrastructure of ECM

Vascular structures were imaged and quantified for differences in abundance, vascular diameter and branch points using a custom MATLAB code (Natick, MA). For analysis of vascular abundance, we identified an area of the fluorescent image, typical of the structure, based on the observation that brighter pixels indicated the presence of structures. The program returned the mean and standard deviation of pixel intensity for the identified structure area. These statistics were subsequently used to set a pixel intensity threshold from which the vascular structure was defined. ImageJ™ was subsequently utilized to measure the percent area covered by each structure. Each image was thresholded and analyzed for percentage of pixels representing the vascular structures as defined using MATLAB. For analysis of vessel diameter and branch points, phalloidin images were converted to a clean binary mask representing the vascular structures. In this bi-level representation, vascular structures, after subtracting background pixels, carry values of 1, while the removed background pixels carry values of 0. To aid in the identification of vessel structures, areas less than 800 pixels were ignored to avoid superfluous noise. The distance between each nonzero and zero pixel in the binary mask was computed by a Euclidean distance transform. Vascular diameters were quantified by multiplying the resulting distance transform matrix, with a logical “skeletal” matrix representing a pixel thick centerline of the vessel architectures. Vascular branch points were enumerated using the “skeletal” depiction of vessel assemblies using built-in MATLAB functions.

For analysis of ECM ultrastructure, SEM images were used. In addition to subtracting the background in the SEM images, a filter was applied to reduce the amount of pixelated noise before processing for fiber analyses. Percent coverage, evaluated as the area of visible pixels to the total area of the binary image representation, was appraised in conjunction with fiber architecture and branch points, as described above for vascular structures.

### ECM quantification

De-cellularized ECM from hypoxia or atmospheric NuFF/MDA231 co-cultures was collected and pooled from three 6 well plates each using previously established methods [8].

### Statistical analyses

All analyses were performed in triplicates for n=3. For vascular image analysis a total of five non-overlapping, representative fluorescent images at 40x magnification were taken per well in each of four wells for a total of 19-20 images/condition analyzed. For ECM ultrastructure analysis, a total of three to four low magnification (10,000 x) SEM images in each of three wells for a total of nine to ten images/condition analyzed. Statistical analysis was performed using GraphPad Prism 4.02 (GraphPad Software Inc., La Jolla, CA). GraphPad Prism 4.02 was used to perform student’s ttests. Significance levels were set at *p ≤ 0.05, **p ≤ 0.01, and ***p ≤ 0.001. All graphical data are reported as the mean ± SD.

## Results

### Experimental set up

**Figure 1:**
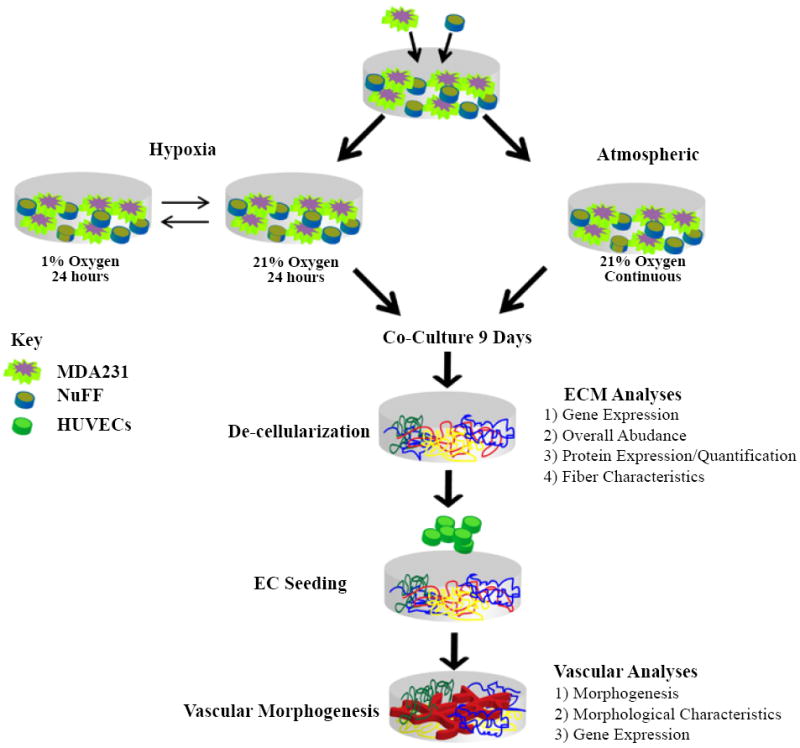
Schematic of culture set up and hypoxia exposure NuFF and MDA231 cells were co-cultured in a 1:1 ratio in atmospheric oxygen (21% O_2_) or alternating 24 hour cycles of hypoxia (1% O_2_) and atmospheric oxygen (21% O_2_) for a period of 9 days. Subsequently, cells were lysed using a strong base and resulting ECM was investigated for changes in protein expression or was utilized as a substrate for HUVECs to form CLS.

Previously, we demonstrate that ECM from NuFF/MDA231, as compared to ECM from NuFF and co-cultures of NuFF with other breast cancer cells, support abundant and functional Capillary-Like Structure (CLS) formation from HUVECs [8]. us, to test the hypothesis that hypoxia affects ECM properties that in turn regulate EC responses and morphogenesis, a NuFF/MDA231 co-culture setup was utilized. We chose to focus our efforts on alternating cycles of 24 hours in hypoxia and 24 hours in atmospheric oxygen. This intermittent hypoxia schedule was important considering the length of time in which co-cultures are maintained (e.g. 9 days), a period necessary for cells to produce a robust monolayer of ECM [8]. Moreover, this approach allowed us to investigate how an intermittent hypoxia schedule, a phenomenon which is present in the tumor microenvironment [32,33] and has been shown to promote angiogenesis [34], influences matrix properties, which in turn, alter vascular formation. While the levels of oxygen experienced by cells cultured in atmospheric conditions are higher than that experienced by oxygenated tumor cells, this approach nonetheless addresses how cells respond to changing environmental stimuli through secretion and deposition of ECM. Using this approach, co-cultures were exposed to 1% O_2_ (hypoxia) for 24 hours, followed by re-oxygenation at 21% O_2_ (atmospheric) for 24 hours over the 9 day culture period (Figure 1). After this time, co-cultures or deposited ECM were investigated for differences in ECM protein expression, deposition and abundance (Figure 1). Deposited ECM was additionally utilized as a substrate from which to investigate vascular morphogenesis (Figure 1). Since we have previously determined that cells experience a ~10 degree temperature drop (from 36°C to 26°C) following chamber flushing (unpublished observations), we took care to note any differences in cell morphology during the course of our hypoxia studies. No overt changes as evidenced by cell rounding and cell de-attachment were observed to occur between atmospheric and hypoxia-grown cells (data not shown).

### Gene expression patterns varied between hypoxia and atmospheric exposed NuFF/MDA2231 co-cultures

**Figure 2:**
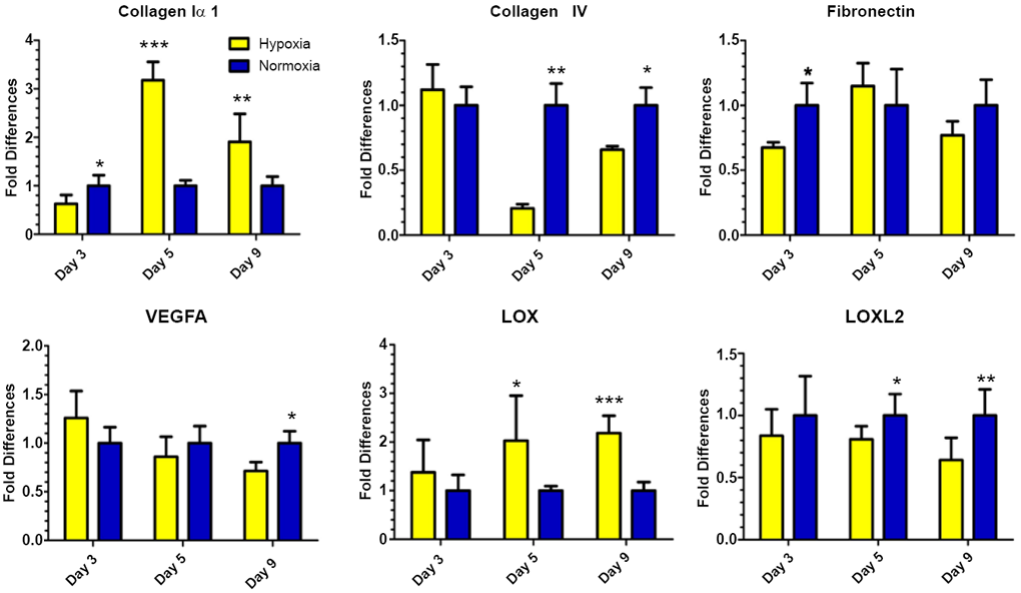
Patterns of gene expression in hypoxia and atmospheric-exposed co-cultures differed during the culture period **(A)** NuFF/MDA231 co-cultures were assessed for changes in the expression patterns of genes important for ECM assembly, stability and angiogenesis along the culture period. Differences were observed between time points for each gene tested. *p≤0.05, **p≤0.01, ***p≤0.001.

We first sought to determine how hypoxia influences changes in the expression of relevant ECM genes in NuFF/MDA231 co-cultures along the culture period. After 3, 5 and 9 days in culture, fibronectin, collagens I and IV, Lysyl Oxidase (*LOX*), Lysyl Oxidase Like-2 (*LOXL2*) and *VEGFA* mRNA expression were quantified in hypoxia and atmospheric-exposed co-cultures. These genes were chosen as they have been shown to be regulated by hypoxia and play an important role in ECM organization and stability and angiogenesis [6, 7, 15-24]. We found that collagen 1αI and LOX mRNA were upregulated in response to hypoxic as compared to atmospheric conditions, while collagen IV, fibronectin, VEGFA, and LOXL2 mRNA were largely downregulated in response to hypoxic as compared to atmospheric conditions (Figure 2).

### Hypoxia-induced changes in ECM protein expression

**Figure 3:**
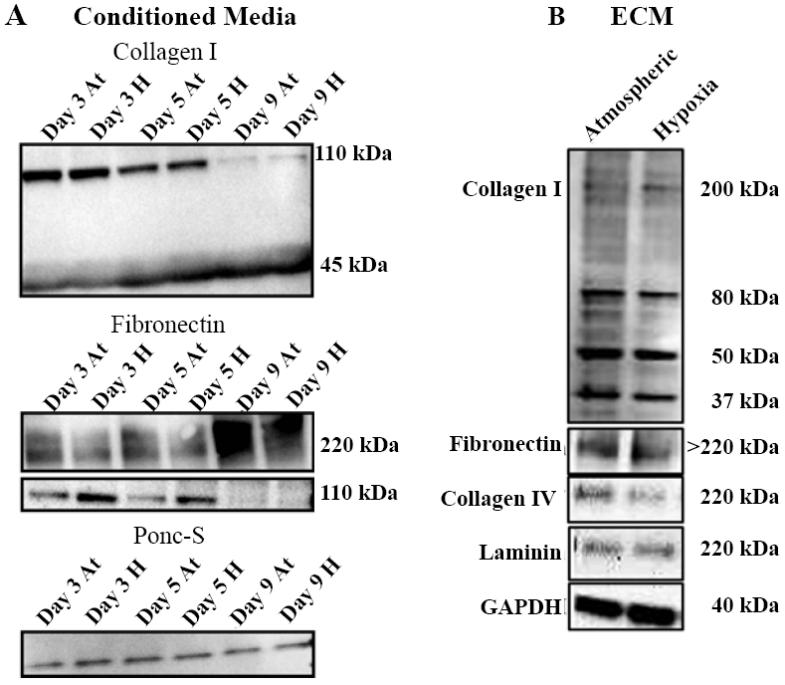
Hypoxia induced changes in ECM protein expression differed depending on the context of culture **(A)** Analysis of collagen I and fibronectin protein expression in the conditioned media of hypoxia and atmospheric-exposed co-cultures. Ponceau S (Ponc-S) was utilized as a loading control **(B)** Western blots of collagen I, fibronectin, collagen IV and laminin in the de-cellularized hypoxic and atmospheric ECM. GAPDH was utilized as a loading control.

We next analyzed co-cultures for the expression of specific ECM proteins. Western blot was utilized to analyze ECM protein expression in cell-conditioned media and de-cellularized matrix from hypoxia and atmospheric-exposed NuFF/MD231 co-cultures. In these studies, deposited ECM from day 9 hypoxic or atmospheric conditions was collected and solubilized. Conditioned media was collected from co-cultures grown in hypoxic or atmospheric conditions for 3, 5 and 9 days, allowing us to better understand secreted ECM protein expression changes occurring during the culture period. Analysis of soluble collagen I and fibronectin, two proteins previously reported to be detectable in cell conditioned media [18], revealed unique trends in protein expression between hypoxia and atmospheric co-cultures. For collagen I, two separate bands were detected in the conditioned media with no obvious differences between hypoxia and atmospheric cultures at respective time intervals. Interestingly, the quantity of the 110 kDa (least processed) fragment of collagen I was decreased in the supernatants of both hypoxia and atmospheric co-cultures during the culture period (Figure 3A). Conversely, the more processed fragment of collagen I (45kDa) was increased in the supernatants of both hypoxia and atmospheric co-cultures along the culture period (Figure 3A). Similar to collagen I, two unique fragments of fibronectin were observed in the conditioned media of hypoxia and atmospheric co-cultures. The 220 kDa full-length fragment was more highly expressed in atmospheric co-cultures during the culture period, with the greatest expression observed at day 9 (Figure 3A). Contrary, the 110 kDa cell binding fragment was more abundant in hypoxia cultures at days 3 and 5, but absent in both hypoxia and atmospheric co-cultures at day 9 (Figure 3A). Within the de-cellularized ECM, few differences in protein expression were observed between hypoxia and atmospheric ECM. The only exception is collagen IV, which was more expressed in atmospheric ECM and the 200 kDa band of collagen I, which was more enriched, albeit slightly, in hypoxic ECM (Figure 3B). GAPDH was utilized as a loading control [8]. No overt differences in expression between atmospheric and hypoxic conditioned were detected (Figure 3B).

### Hypoxia altered fiber morphology and characteristics

**Figure 4:**
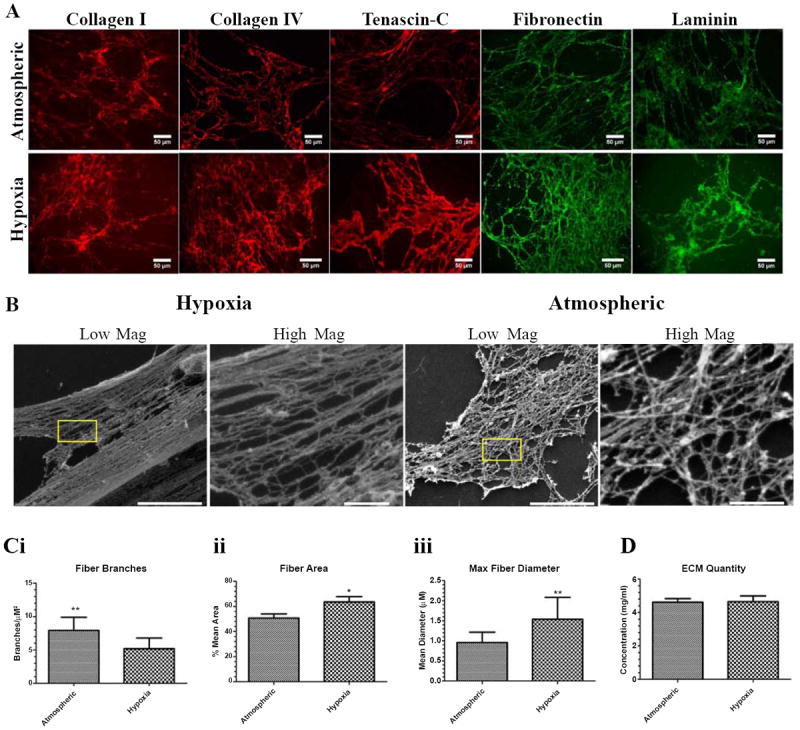
Hypoxia altered the morphology and characteristics of de-cellularized ECM **(A)** ECM deposited from day 9 hypoxic and atmospheric-exposed co-cultures was observed using immunofluorescence for fibronectin, collagens I and IV, laminin and tenascin-C. **(B)** SEM images depict the ultra-structural morphology of hypoxic and atmospheric ECM in low (left) and high (right) magnifications. Scale bars are 5μM and 1μM for low and high magnification images, respectively. **(C)** Quantification of fibers for differences in **(i)** branches, (**ii**) area, and (**iii**) maximum diameters in hypoxic and atmospheric ECM. **(D)** Total ECM concentration in hypoxic and atmospheric ECM. *p≤0.05, **p≤0.01, ***p≤0.001.

As our aim was to determine the direct effect of deposited ECM on vascular morphogenesis, we next analyzed the de-cellularized ECM. In order to obtain de-cellularized ECM, hypoxic and atmospheric co-cultures were treated with a buffer containing a strong base plus a detergent, methodology others and we have previously utilized to obtain cell free matrices [8,9]. Immunofluorescence analyses illustrated the presence of ECM proteins in the de-cellularized matrix of day 9 hypoxia and atmospheric-exposed co-cultures without detectable differences in organization (Figure 4A). Analysis of the ultra-structural characteristics of the de-cellularized ECM revealed that hypoxic ECM contained fibers which were more aligned and compact than their atmospheric ECM counterparts (Figure 4B). As an indication of fiber compaction, we measured the branch density, assessed as the number of fiber branches per μM^2^. In this analysis, we reasoned that as the degree of fiber compaction increases, there would be a corresponding decrease in fiber branch point density. Indeed, fibers in hypoxic ECM possessed significantly fewer branch points than fibers deposited by atmospheric co-cultures (Figure 4Ci). Quantification of additional fiber characteristics revealed that the hypoxic ECM contained a significantly higher percent area of fibers Figure 4Cii) and possessed fibers with significantly higher maximum fiber diameters (Figure 4Ciii). Since evaluation of total deposited ECM, assessed using a colorimetric assay for total protein expression, revealed no differences in ECM quantity (Figure 4Civ), the morphological differences between hypoxia and atmospheric ECM are most likely attributed to the ECM organization.

### Hypoxic ECM did not alter the morphology of CLS

**Figure 5:**
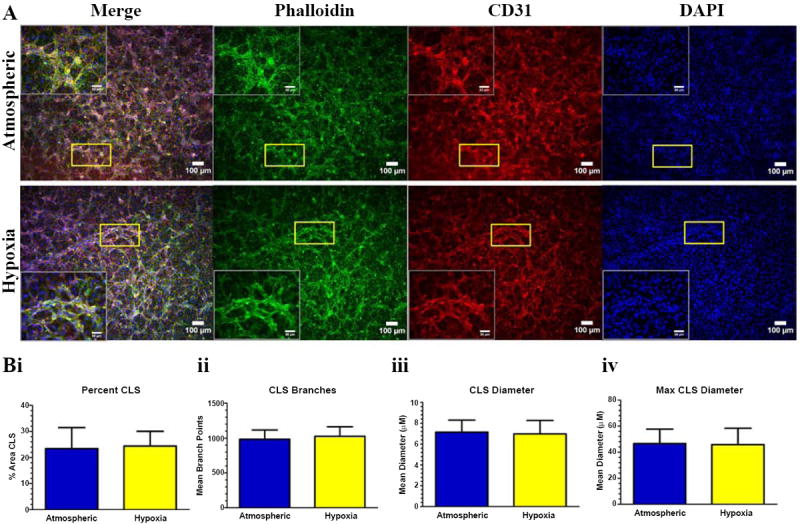
Vascular morphogenesis and morphology were not altered on hypoxic ECM **(A)** Immunofluorescence images of CLS formed from HUVECs cultured atop de-cellularized ECM from hypoxic and atmospheric-exposed NuFF/MDA231 co-cultures. Insets are high magnification of the boxed area. Scale bars are 50 μM for image insets. Phalloidin in green, CD31 in red and nuclei in blue. **(B)** Analyses of vascular morphological features including **(i)** vascular abundance, **(ii)** mean vascular branches, **(iii)** mean vascular diameters and **(iv)** maximum vascular diameters between structures grown on hypoxic and atmospheric ECM.

To elucidate the effect of the hypoxic ECM on vascular morphogenesis, HUVECs were seeded on the de-cellularized hypoxic or atmospheric ECM deposited from NuFF/MDA231 co-cultures. After a period of 24 hours, resulting vascular structures were assessed for differences in overall abundance and morphology (e.g vascular branches and vascular thickness). Representative images of vascular structures grown on hypoxic and atmospheric ECM are shown in Figure 5A. Evidence of vascular lumens was observed in reconstructed z stacks from both conditions, validating the 3-dimensional (3D) nature of the structures. Vascular structures were subsequently assessed for differences in abundance, branching and tube diameters. Quantification of vascular structures revealed no significant differences in overall abundance, analyzed as percent coverage of vascular structures (Figure 5Bi). In addition, differences in the number of vascular branches (Figure 5Bii) and mean (Figure 5Biii) and maximum vascular diameters (Figure 5Biv) were not observed. In order to confirm that these results were not due to cell culture affects, we analyzed vascular morphogenesis on ECM deposited from hypoxia and atmospheric-exposed NuFF monocultures (i.e. in the absence of MDA231 breast cancer cells). Similar to NuFF/MDA231, ECM from hypoxia and atmospheric-exposed NuFF did not promote detectable differences in vascular morphogenesis (data not shown).

### Hypoxic ECM regulated the expression of angiogenic growth factors and MMPs in CLS

**Figure 6:**
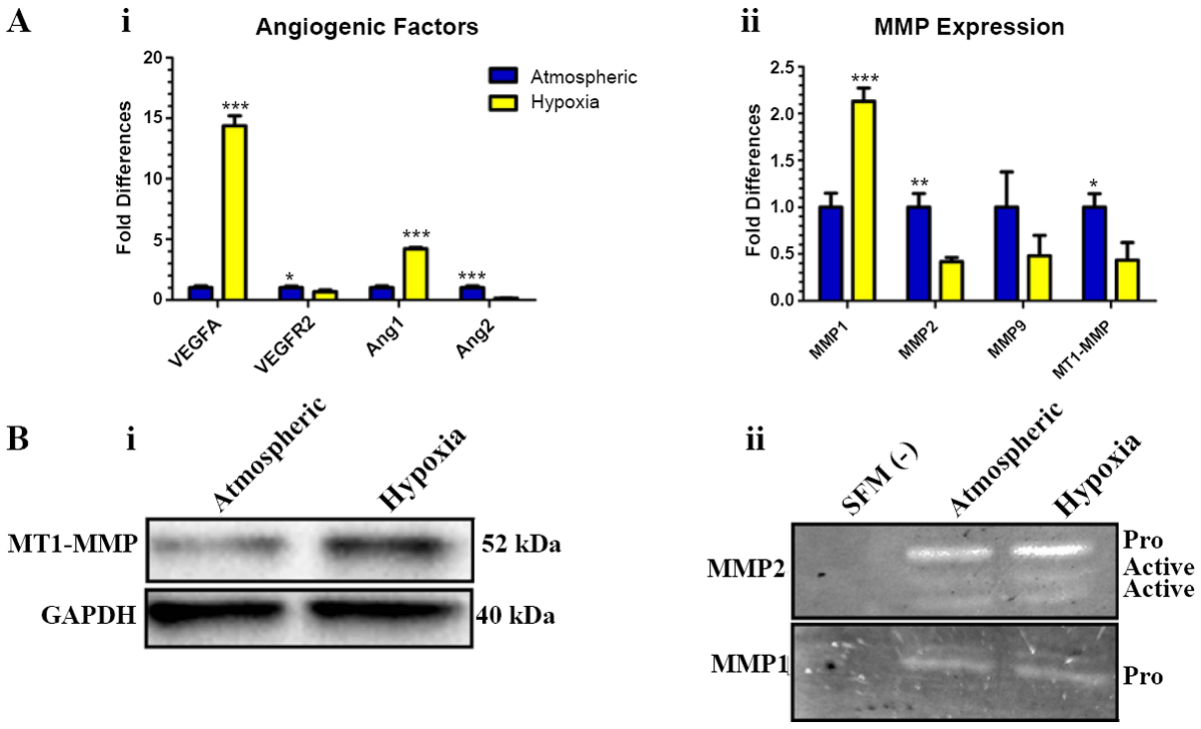
Pro-angiogenic factors and MMPs were differentially expressed in CLS grown atop hypoxic and atmospheric ECM **(A)** qRT-PCR for **(i)** VEGFA, VEGFR2, Ang1, Ang 2 and **(ii)** MMPs 1,2, and 9 and MT1-MMPin CLS grown on hypoxic and atmospheric de-cellularized ECM. **(B) (i)** Western blot for MT1-MMP and **(ii)** zymography for MMP1 and 2 in CLS grown on hypoxic and atmospheric ECM. Serum free media (SFM) was used as a control. *p≤0.05, **p≤0.01, **p≤0.001.

To analyze the effect of hypoxic ECM on ECs, we next examined differences in gene expression of several pro-angiogenic factors and MMPs. While hypoxia regulates the expression of a number of proteins, we elected to narrow our focus to those factors, which play a prominent role in angiogenesis. Evaluation of additional hypoxia-induced factors was beyond the scope/interest of our present investigation. Utilizing qRT-PCR, we found that VEGFA and Ang1 were significantly upregulated while Ang2 and VEGFR2 were significantly down-regulated in vascular structures grown on hypoxic ECM compared to those grown on atmospheric ECM (Figure 6Ai). Regarding MMP expression, we elected to investigate MMPs 1, 2, 9 and MT1-MMP, chosen for their known roles in angiogenesis [30,35-38]. MMP1 mRNA was upregulated in CLS grown on hypoxic ECM while MMPs 2 and MT1-MMP mRNA were downregulated compared to CLS grown on atmospheric ECM (Figure 6Aii). Since differences in MMPs were observed at the mRNA level, we elected to investigate protein expression and protease activity of these. Interestingly, the expression of MT1-MMP was higher for CLS grown on hypoxic as compared to atmospheric ECM (Figure 6Bi) while zymography did not reveal any differences in proteolytic activity for MMPs 1 and 2 between CLS grown on hypoxic and atmospheric ECM (Figure 6Bii).

## Discussion

Hypoxia, a ubiquitous presence in the tumor environment and participant in tumor progression, is known to alter gene and protein expression profiles of a number of ECM proteins [15-24], many of which have known roles in angiogenesis [6,7]. While numerous groups have independently identified hypoxia-induced alterations in the expression patterns of ECM genes and in patterns of angiogenesis, none have investigated whether hypoxia-induced matrix abnormalities perturb vascular morphogenesis. Inspired by our previous study in which we report breast cancer matrix-induced differences in vascular morphology, we sought to determine whether hypoxia influenced breast tumor matrix properties, which in turn altered vascular behaviors.

Hypoxia has been well-documented to influence the expression of several ECM genes and proteins [15-24], the best characterized being collagen I, and modifiers of matrix stability, such as LOXs [16-20,25]. Our studies support a role for hypoxia in altering gene expression in that we observed significant up-regulation of collagen Iα1 and LOX expression in co-cultures subjected to hypoxia. Despite this change in collagen Iα1 gene expression, hypoxia-induced increases in the expression of collagen I in the conditioned media of co-cultures along the culture period were not detected. An interesting trend was nonetheless observed in collagen I expression in the conditioned media of co-cultures in that the expression of the larger, least processed fragment steadily decreased along the culture period until it was almost undetectable after 9 days of hypoxic or atmospheric culture. This steady decline may point to the likelihood of collagen I being assembled into the matrix as its expression was highly detected in the de-cellularized ECM deposited from both day 9 hypoxic and atmospheric co-cultures. Interestingly, a slight increase in the larger, least processed fragment of collagen I was detected in the de-cellularized hypoxic ECM, potentially suggesting that an as yet unidentified hypoxia-induced mechanism may be responsible for the increased assembly of this fragment in the matrix. Regarding fibronectin, it is possible, given that the 220 kDa and 110 kDa fragments were increased in atmospheric and hypoxic conditioned media, respectively, that hypoxia-regulated differences in overall fibronectin expression may not be evident in the de-cellularized ECM. Given that distinct changes in mRNA and ECM protein expression were observed and were dependent on the context evaluated (e.g. gene expression versus protein expression in conditioned media and de-cellularized ECM), these data should warrant caution in the choice of an endpoint for analyzing hypoxia-mediated effects on matrix gene and protein expression.

Hypoxia has been reported to influence matrix organization and compliance, promoting greater fiber alignment and matrix stiffness [18]. Similarly, our ultra-structural analyses of matrix organization revealed that hypoxia induced a greater degree of fiber organization, apparent as hypoxic fibers were more compact and aligned than their atmospheric counterparts. In addition, hypoxic fibers occupied a greater percent area and possessed larger diameter fibers than those deposited by co-cultures maintained in atmospheric conditions. Although we did not investigate mechanical stiffness in these matrices, it is conceivable, given its degree of organization and compaction in addition to the up-regulated expression of the matrix stiffening enzyme LOX [39] in hypoxic co-cultures, that the hypoxic matrix would be stiffer than the matrix deposited by atmospheric co-cultures. Since changes in overall ECM abundance, measured using a colorimetric assay for total protein expression, did not differ and protein expression differences in the de-cellularized matrix were negligible, it is likely that the changes in fiber organization are related to activation of hypoxia-responsive gene(s). Although this gene/gene signature has not yet been identified, pathways activated by hypoxia inducible factor 1 alpha (HIF-1α), the oxygen-responsive subunit of HIF, are implicated as it has recently been shown that fiber alignment was abrogated in hypoxia-treated fibroblasts when HIF-1α was knocked down [18]. A greater understanding of hypoxia-induced effects on fiber organization is imperative, especially in the context of breast cancer as a stiffened stroma is not only a prognostic indicator of tumor formation, but promotes breast tumor growth, invasion and metastasis [39-41]. As such, in future studies it will be important to track the expression of HIF-1α during the course of the experiments, modifying its expression through gain and loss of function in NuFF and MDA231 to elucidate its role in fiber morphology. Overall, our studies demonstrated that hypoxia directed morphological changes in fiber organization in the de-cellularized breast tumor matrix, findings which may have important implications for dissecting hypoxia-induced matrix changes on malignant cell behaviors.

Given the morphological features of the hypoxic ECM in addition to recent reports implicating the topography of the matrix in angiogenic responses [42-45], we reasoned that vascular morphogenesis and morphology may be altered in ECs cultured atop of the hypoxic matrix. Surprisingly, neither vascular morphogenesis nor morphology, as investigated from the percent total CLS in addition to mean CLS diameter and branch points, did not significantly differ between CLS grown atop hypoxic and atmospheric ECM from both co-cultures and monocultures of NuFF alone. The absence of vascular morphological differences is surprising especially given hypoxia’s well-known role in activating angiogenesis. However, it is important to consider that these *in-vitro* studies have investigated hypoxia-induced effects on angiogenesis through evaluation of EC response to hypoxia [46,47]. Our studies are unique in that we have investigated EC response to hypoxic ECM. The fact that we did not observe morphological changes in ECs cultured atop the ECM may indicate that the period in which we conducted our analyses (e.g. 24 hours) was not suitable for the detection of distinct morphological differences. Indeed, this may be one constraint of the system as we and others have found optimal vascular network formation on de-cellularized ECM to occur at 24 hours [8,9], with longer periods resulting in vascular disassembly and shorter periods resulting in less robust vascular network formation. In the future, it will be important to address whether vascular kinetics (e.g. adhesion, migration, and time to vascular assembly), evaluated over the entire 24 hour period of vascular network formation, are altered on hypoxic ECM. In addition, it will be important to address whether these observations are unique to MDA231 or whether they extend to less malignant breast cancer cells and/or other tumor cell lines (e.g. colon, prostate, etc).

Despite the absence of differences in vascular morphogenesis and morphology, we observed that the hypoxic ECM nonetheless promoted up-regulated expression of vascular pro-angiogenic factors VEGFA and Ang1, down-regulation of the vascular destabilizing factor Ang 2 and up-regulation of proteolytic enzymes MT1-MMP and MMP1, suggesting that the hypoxic ECM does indeed activate angiogenic responses. While VEGFA is a well-known, hypoxia-regulated mitogenic factor [48], Ang1 has not, to our knowledge, been previously reported to be activated by hypoxia in ECs. Similarly, MT1-MMP has previously been reported to be up-regulated in ECs in response to hypoxia [47], but MMP1, to our knowledge, has not. Regardless, the observed changes in gene and protein expression levels of these factors suggest a novel mechanism wherein the hypoxic ECM exerts effects on angiogenic responses. Although the exact mechanism is at present unknown, it is possible that the greater degree of fiber organization in the hypoxic ECM will play a role. For instance, fiber organization has been reported to accompany matrix stiffness [18] and matrix stiffness, in turn, has been reported to enhance angiogenic sprouting and invasion into the matrix [49]. The ability of vascular structures to invade the matrix and form vascular structures is dependent on vascular expression of MMPs [27], with vascular sprouting and survival occurring in response to VEGF and Ang1 [50,51]. In this manner, it is conceivable that matrix-induced changes in angiogenic factors and MMPs may provide a network-forming advantage to these ECs early on during vascular development. Another possibility is that the matrix may induce constitutive up-regulation of these factors in vascular structures, so that, in the presence of an angiogenic stimulus, they will be primed to quickly form new structures. These possibilities are intriguing and will require additional investigation in order to better understand the hypoxic matrix-induced effects on angiogenic responses.

In conclusion, we report for the first time that the hypoxic ECM alters EC responses, namely expression of pro-angiogenic and proteolytic factors. These differences may be attributed to alterations in ECM ultrastructure, such as fiber morphology, as the hypoxic ECM possessed fibers that were more compact, aligned, and larger in diameter. Thus, we speculate that one mechanism whereby the hypoxic ECM may participate in the regulation of angiogenic responses is through alterations in fiber architecture. We recognize that the oxygen concentration utilized for our control cultures (e.g. atmospheric) is much higher than that which is present in the body. As such, additional studies employing oxygen concentrations, which had better recapitulate the host tissue environment, will be necessary to determine whether differences reported herein exist when physiological oxygen concentrations are utilized as a control. Given the profound role of hypoxia in tumor progression, further understanding of the implications of the hypoxic ECM on angiogenesis and vascular cell responses will be imperative for addressing the diverse roles of hypoxia in tumorigenesis.

## Figures and Tables

**Table 1:** Breast cancer cell lines.

Antibody	Concentration	Vendor
Fibronectin	1:200 IF; 1:200 WB	Sigma
Tenasin-C	1:50 IF	Santa Cruz
Collagen I	1:250 IF 1:1,000 WB	Abcam; NIDCR
Collagen IV	1:100 IF; 1: 250 WB	Santa Cruz; Abcam
Laminin	1:100 IF; 1:500 WB	Abcam
MT1-MMP	1:1,000 WB	Abcam
GAPDH	1:3,000 WB	Cell Signaling Technology
CD31	1:200 IF	Dako
Phalloidin	1:200 IF	Molecular Probes
Alexafluor FITC	1:1,000 IF	Invitrogen
Cy3	1:100 IF	Sigma
FITC	1:100 IF	Sigma
HRP anti-rabbit	1:1,000 WB	Cell Signaling Technology
HRP anti-mouse	1:3,000 WB	Cell Signaling Technology

IF: Immunofluorescence; WB: Western Blot

## Abbreviations

Ang1: Angiopoietin 1
Ang2: Angiopoietin 2
CLS: Capillary-Like Structure
EC: Endothelial Cell
ECM: Extracellular Matrix
HIF-1α: Hypoxia Inducible Factor 1 α
HUVECs: Human Umbilical Vein Endothelial Cells
LOX: Lysyl Oxidase
LOXL2: Lysyl Oxidase-Like 2
NuFF: Neonatal Foreskin Fibroblasts
MMP: Matrix Metalloproteinase
SEM: Scanning Electron Microscopy
VEGFA: Vascular Endothelial Growth Factor A
VEGFR2: Vascular Endothelial Growth Factor Receptor 2
